# Harnessing Artificial Intelligence to Predict Dental Crowding in Modern Orthodontic Practice: A Comprehensive Review

**DOI:** 10.1002/kjm2.70304

**Published:** 2026-10-15

**Authors:** Xiwen Xin, Shun‐Yu Kan, Tianle Chen, Ching‐Chang Ko

**Affiliations:** ^1^ Division of Orthodontics The Ohio State University College of Dentistry Columbus Ohio USA; ^2^ Department of Computer Science Boston University Boston Massachusetts USA

**Keywords:** artificial intelligence, dental crowding, digital orthodontics, intraoral scanning, PointNet++

## Abstract

Dental crowding is fundamental to orthodontic diagnosis and treatment planning, yet what constitutes “crowding” remains surprisingly heterogeneous. Space analysis quantifies tooth size–arch length discrepancy, irregularity indices characterize alignment displacement, and clinician‐assigned severity reflects broader diagnostic interpretation. This narrative review examines the evolution of crowding assessment from conventional space analysis and digital measurement to two‐dimensional artificial intelligence (AI), three‐dimensional point‐cloud learning, and clinician‐aware agentic AI. Digitalization improves efficiency and reproducibility but does not resolve fundamental dependencies on how crowding is defined, anatomically represented, and established as a reference standard. Two‐dimensional models enable prediction from readily available images but are constrained by projection and scale, whereas three‐dimensional approaches directly learn from dental surface representations and can capture local geometric relationships. In a companion study of 495 arches from 284 patients, PointNet++ achieved an *R* ^2^ of 0.718 and a mean absolute error (MAE) of 1.54 mm for prediction of crowding. In a separate retrospective demonstration, a bounded Gemini‐based Agent incorporating matched record‐derived verbal information reduced MAE from 1.951 to 1.437 mm, whereas mismatched information degraded performance. These findings also illustrate that the target construct—the specific clinical feature of crowding that a model is trained to predict—may differ across assessment methods. Together, these findings highlight a fundamental challenge: improving prediction does not resolve ambiguity in the target construct. Future crowding AI should therefore align the target construct, reference standard, and input representation; preserve provenance and uncertainty; integrate complementary clinical information transparently; and route uncertain cases for expert review.

## 1 Introduction

Dental crowding is among the most prevalent malocclusions encountered in orthodontic practice and remains an important consideration in orthodontic diagnosis and treatment planning. Accurate assessment of crowding is clinically important because both the magnitude and distribution of space discrepancy can influence treatment objectives, extraction decisions, biomechanics, and retention planning. One conventional approach quantifies crowding as the discrepancy between cumulative mesiodistal tooth width and available arch space, commonly expressed as tooth size–arch length discrepancy (TSALD) [1]. However, the apparent simplicity of this measurement can obscure the broader clinical complexity of crowding. Crowding is not a single, uniformly defined clinical entity, but rather reflects the interplay among tooth size and morphology, arch dimensions and form, dentoalveolar and periodontal boundaries, and the position and orientation of individual teeth within the dental arch. Consequently, different measures of crowding may capture different aspects of the same malocclusion, and both the measured magnitude and its clinical interpretation may vary according to what aspect of crowding is being assessed, how it is measured, and what reference standard is used.

## 2 What Does Dental Crowding Measure?

### 2.1 Tooth Morphology and Arch Form

Crowding may result from a discrepancy between tooth size and available dental arch dimensions. Early orthodontic evaluations of crowding were based largely on qualitative clinical observation and visual estimation of tooth alignment. During the early development of orthodontics, clinicians emphasized arch form, dental alignment, tooth position, and occlusal relationships as essential components of diagnosis [2]. Although clinically useful, such assessments were inherently subjective and could vary with clinician experience, visual judgment, and treatment philosophy. As orthodontics evolved toward a more measurement‐based specialty, increasing attention was directed toward quantifying the relationship between tooth material and available arch dimensions, marking the beginning of modern orthodontic space analysis.

Among the earliest quantitative approaches, Lundström emphasized that malocclusion should be interpreted in relation to the apical base and underlying supporting skeletal structures, rather than solely as a problem of crown alignment [3]. Nance subsequently formalized mixed‐dentition space analysis by comparing the space required by the dentition with the space available within the dental arch [4]. In these traditional analyses, mesiodistal tooth widths were measured on plaster casts using calipers, while available arch length was estimated using brass wire, dividers, or segmented arch measurements. The discrepancy between available and required space provided a numerical estimate of space deficiency or excess, with negative values indicating crowding and positive values indicating spacing when calculated as available minus required space (Equation (1); Figure 1). This shift from visual judgment to measurable space discrepancy was an important step in the development of crowding assessment, establishing the principle that crowding could be quantified rather than described solely as irregular tooth alignment.

(1) TSALD=available arch space−required tooth space

**FIGURE 1:**
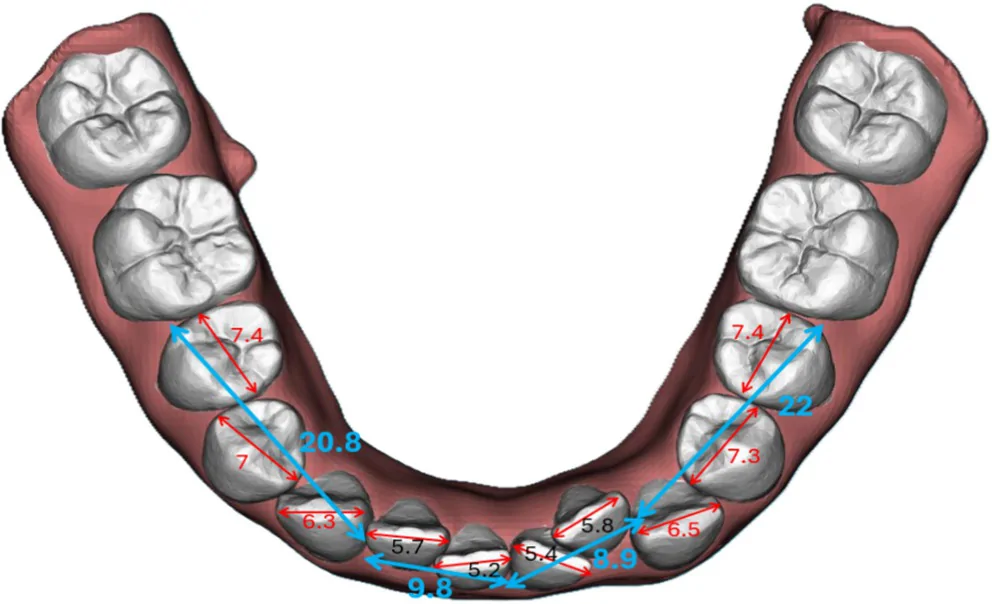
TSALD diagram. Space available is defined as the arch length from the mesial surface of one first permanent molar to the mesial surface of the contralateral first permanent molar (blue). Space required represents the total mesiodistal width (red) of the teeth to be accommodated within this arch segment. The comparison between the available space and space required reflects the degree of crowding (negative value) or spacing (positive value) within the dental arch.

The clinical value of early space‐analysis methods was substantial because they allowed clinicians to evaluate treatment options more systematically, including arch expansion, molar distalization, interproximal reduction, serial extraction, or premolar extraction. However, important limitations remained because TSALD values depend on how available arch space is defined and measured. In the digital dentistry era, various geometric models and mathematical functions have been proposed to represent the human dental arch, typically requiring identification of anatomical landmarks for curve fitting. The resulting curve can then be used to estimate arch length or perimeter. Nevertheless, landmark selection remains operator‐dependent because the chosen reference points may vary in the buccolingual, occlusogingival, and mesiodistal dimensions of three‐dimensional space.

Conventionally, landmarks may be placed at the buccal cusp tips of posterior teeth and the midpoints of the incisal edges in a setup (aligned) dentition. A curve fitted through these landmarks can be used to define the arch form and estimate the arch perimeter for TSALD assessment. Andrews questioned dental arch forms derived solely from tooth‐based landmarks because they may not adequately account for the underlying alveolar process. Instead, he proposed the WALA ridge as an anatomical reference for individualized arch form. The WALA ridge is a band of soft tissue near the mucogingival junction of the mandibular alveolar process. He proposed that this anatomical reference could guide a stable, individualized arch form for tooth positioning and potentially provide a biologically informed basis for estimating available arch space [5] (Figure 2).

**FIGURE 2:**
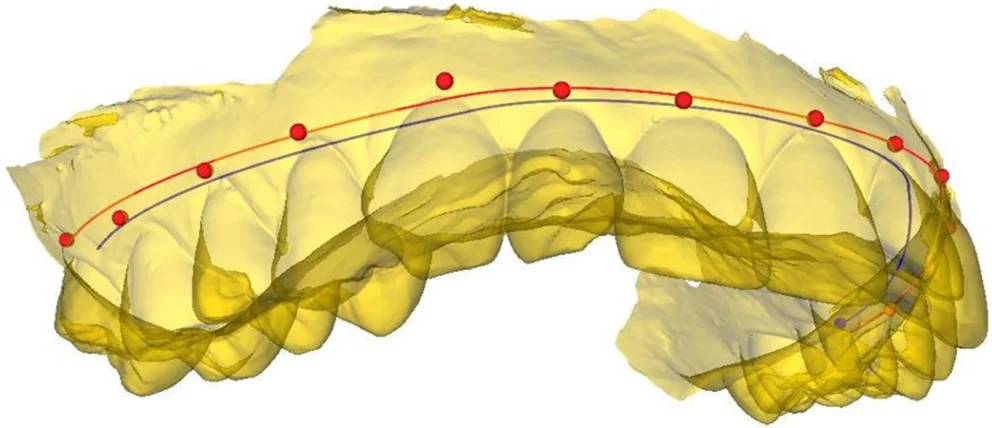
Individualized dental arch form based on the WALA ridge.

Beyond landmark selection, the way the dental arch is mathematically described introduces another source of variability. Dental arch form can be characterized by parameters such as arch width, arch depth, intercanine width, and intermolar width. Various mathematical and computational approaches, including polynomial, catenary, beta‐function, vector‐quantization, and other parametric representations, have been applied to characterize dental arch form [6, 7, 8, 9, 10, 11, 12, 13, 14]. Such mathematical models may provide a more individualized basis for estimating arch perimeter and, consequently, TSALD.

However, a more precise mathematical description does not necessarily provide a more complete description of crowding. These methods still reduce a complex three‐dimensional arrangement of teeth and supporting structures to a limited set of linear measurements or an idealized arch curve. Features that are clinically relevant to crowding—including tooth rotation, buccolingual displacement, crown‐root axial inclination, arch curvature, alveolar morphology, and skeletal relationships—may be only partially captured or not captured at all. Consequently, even when crowding is measured quantitatively, the resulting value reflects the assumptions built into how the dental arch is defined and measured. In this sense, quantitative space analysis improves measurement precision without necessarily making the assessment entirely objective.

### 2.2 Alignment Irregularity Index

In 1975, the introduction of Little's Irregularity Index represented another important milestone in the evolution of crowding assessment [15]. The primary purpose of Little's Index was to provide a simple and reproducible quantitative method for assessing mandibular anterior alignment irregularity, thereby reducing reliance on subjective clinical descriptions such as mild, moderate, or severe crowding. Unlike TSALD analysis, Little's Index does not measure generalized arch‐length discrepancy; instead, it quantifies mandibular anterior alignment irregularity by summing the linear displacement between adjacent anatomical contact points of the six mandibular anterior teeth. Because of its simplicity and reproducibility, it became widely used in studies evaluating mandibular anterior relapse and post‐treatment stability. Nevertheless, Little's Index also highlights a fundamental conceptual issue in orthodontics: different methods used to assess “crowding” may quantify distinct clinical constructs. TSALD estimates generalized space discrepancy, whereas Little's Index primarily measures anterior contact‐point displacement. A patient with one severely displaced incisor may exhibit a high irregularity score despite minimal overall arch‐length discrepancy, whereas another patient with generalized space deficiency may not be adequately represented by anterior irregularity alone. Thus, the term “crowding” may obscure the fact that different measurement systems evaluate fundamentally different clinical characteristics (Figure 3).

**FIGURE 3:**
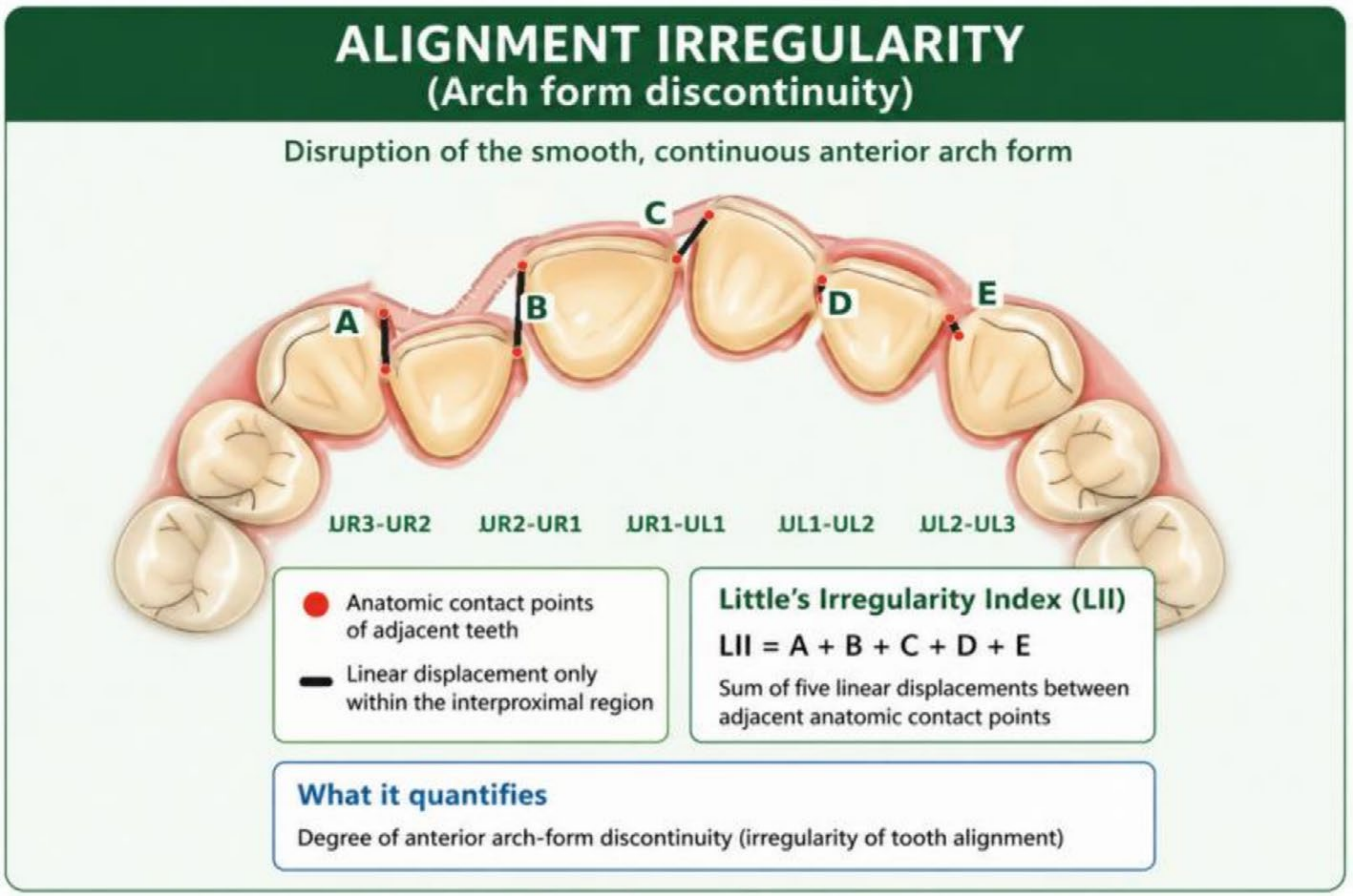
Irregularity index.

### 2.3 Etiology and Clinical Interpretation

The biological etiology of dental crowding has also been extensively investigated. Previous studies have demonstrated significant relationships among tooth size, arch dimensions, and dental crowding [1, 16, 17, 18, 19]. Collectively, these investigations suggest that crowding is not determined by a single factor, but rather by interactions among tooth size and arch dimensions. These multifactorial relationships are clinically relevant because crowding cannot be interpreted solely as a quantitative space discrepancy.

Consequently, the same measured TSALD may be associated with substantially different treatment decisions depending on clinician philosophy and patient‐specific characteristics. Tom et al. recently demonstrated that crowding estimation and extraction recommendations varied considerably among orthodontists, highlighting the substantial interpretive variability inherent in orthodontic diagnosis [20]. These findings reinforce a central conceptual issue in orthodontics: dental crowding assessment is not merely a measurement problem, but also a problem of diagnostic interpretation.

## 3 Quantification Methods of Crowding

### 3.1 From Manual Models to Digital Orthodontics

Traditional orthodontic space analysis relied on plaster casts, calipers, brass wires, dividers, and manually selected landmarks. The transition to digital dentistry in the late twentieth and early twenty‐first centuries fundamentally changed how orthodontic records could be captured, measured, and compared. Digital dental models and intraoral scanners have replaced many of these steps by providing digital representations of the dentition that facilitate standardized and reproducible measurements. Digital workflows allow repeated measurements of tooth width, arch dimensions, Bolton discrepancy, and space analysis, while also facilitating data storage, communication, virtual setup, appliance design, and longitudinal comparison [21, 22, 23, 24, 25, 26, 27, 28, 29, 30, 31]. Computer‐assisted orthodontic software systems such as OrthoCAD and 3Shape OrthoAnalyzer have been used for this purpose. Multiple studies demonstrated that digital orthodontic models provide clinically acceptable accuracy and reproducibility for diagnostic measurements, although variability may still occur depending on crowding severity and measurement complexity [21, 22, 23, 24, 25, 26, 27]. Nevertheless, discrepancies between software‐derived measurements and clinical assessment may occur, particularly in complex cases, underscoring the continued need to interpret digital measurements together with direct clinical and anatomical findings.

Importantly, digitalization changed how crowding could be measured, but it did not eliminate the underlying challenges in how crowding is defined and quantified. A digital dental model still requires the clinician or measurement system to define which landmarks to use, how available space should be estimated, and which anatomical boundaries should be considered relevant. Different curve‐fitting methods may produce different arch lengths or perimeters. Similarly, a rotated tooth may be interpreted as a local tooth‐position problem, a difference in how available space is distributed among the teeth and how that distribution contributes to crowding, or a manifestation of a broader arch‐form discrepancy. Thus, greater measurement reproducibility does not automatically translate into greater agreement about the clinical meaning of crowding. Digital models improve the consistency and efficiency of measurement, but the clinical interpretation remains dependent on how the dentition is defined, measured, and understood.

Artificial‐intelligence‐based approaches extend digital orthodontics from measuring predefined features to learning patterns directly from dental records. Once a dental arch is stored as an image, mesh, or point cloud, a computational model can learn associations between morphology and a target label. The central question is then not only whether the model predicts accurately, but also which target construct is being predicted and whether the input representation preserves the anatomical information required to make that prediction.

### 3.2 Artificial Intelligence (AI) for Crowding Assessment

AI‐based approaches extend digital orthodontics by allowing computers to learn patterns from dental images and three‐dimensional dental models rather than relying entirely on manually defined measurements. In orthodontics, AI has been applied broadly to diagnosis and treatment‐planning tasks, including extraction‐decision prediction [32, 33, 34, 35, 36, 37, 38], and more recently to direct crowding assessment [39, 40, 41]. For crowding assessment, inputs may include intraoral photographs or three‐dimensional scan‐derived representations, while outputs may include a numerical estimate of space discrepancy in millimeters or a categorical assessment of crowding severity [39, 40, 41]. These choices are clinically important because they determine both what aspects of the dentition are available for assessment and what clinical question the AI system is actually answering.

The two output types are related but not interchangeable. A numerical prediction provides a continuous estimate and can distinguish differences within the same severity range, whereas classification places a patient into predefined severity categories. Classification may be convenient for screening and communication, but its result depends on how the boundaries between categories are defined and may change when a prediction falls close to a category boundary. Neither approach is free from these underlying assumptions: both numerical and categorical predictions ultimately depend on the measurement or clinical assessment used as the reference. Thus, even when an AI system closely reproduces the measurement or clinical assessment used for training and evaluation, it remains unclear whether this necessarily provides a more complete representation of the clinical condition itself.

This review examines how different AI approaches have been applied to crowding assessment and how their choices of input and output affect the clinical interpretation of their predictions. Among these approaches, two‐dimensional convolutional neural networks (CNNs) and three‐dimensional point‐based neural networks represent two important predictive strategies. Selected studies are used not only to summarize their reported performance but also to illustrate what information each approach can capture and what may be lost in the process [39, 40, 41]. The principles of agentic AI are then examined as a potential next step for dental applications. Rather than replacing individual prediction models, agentic AI may provide a framework for bringing together complementary clinical information—such as dental morphology, measurements, photographs, and clinical records—and using that information to support a more context‐aware assessment. This progression raises a broader question for crowding assessment: as AI becomes increasingly capable of combining and interpreting clinical information, how can we ensure that it is predicting the clinical condition we intend to measure, rather than simply reproducing the assumptions and limitations of the measurements or labels used to train and evaluate it?

#### 3.2.1 Two‐Dimensional Image Models

Two‐dimensional image models represent one of the earliest applications of AI to orthodontic assessment, using photograph images to identify patterns associated with dental crowding. A convolutional neural network (CNN) processes an image through a series of layers that progressively identify increasingly complex visual features. Early layers detect simple features such as edges and changes in brightness, while deeper layers combine these features to recognize tooth contours, contact areas, and overall patterns of dental alignment. Pooling or other image‐reduction operations allow later layers to consider information from progressively larger areas of the image.

VGG networks implement this approach using sequential stacks of small 3 × 3 convolutional filters and pooling operations [42]. Their relatively uniform structure makes the progression of image processing straightforward, although information is passed through each layer in sequence. ResNet introduced shortcut connections that allow information to bypass selected processing layers [43]. Rather than requiring each layer to learn an entirely new representation, the network can learn additional changes to the information already identified, facilitating the training of deeper networks.

For crowding assessment, the final output of the network determines what clinical question it answers. A regression output provides a continuous estimate of crowding, such as a space discrepancy in millimeters, whereas a classification output assigns the patient to predefined severity categories. Accordingly, model performance should be interpreted in relation to the type of clinical assessment being produced. Reporting both the numerical error in millimeters and agreement with clinical severity grades can help distinguish how closely a model estimates the measured amount of crowding from how consistently it identifies the clinically defined severity category.

Ryu et al. evaluated VGG16, VGG19, ResNet50, and ResNet101 using 3136 orthodontic occlusal photographs to perform landmark detection, crowding categorization, and extraction diagnosis [40]. VGG19 showed the highest agreement for crowding categorization, with a Cohen's weighted κ of 0.73 for both the maxilla and mandible, indicating substantial agreement with the reference ratings beyond chance. The same architecture achieved landmark‐detection mean errors of 0.84 and 1.06 mm for the maxilla and mandible, respectively.

The main limitation of a photograph is that it projects three‐dimensional anatomy onto a plane. Tooth rotation, torque, buccolingual displacement, arch depth, and interproximal relationships may be hidden or may produce similar two‐dimensional appearances. Perspective, mirror angulation, lighting, and the lack of calibrated scale add further uncertainty. A CNN can infer some depth‐related cues, but the missing geometry is not explicitly present in the input (Figure 4).

**FIGURE 4:**
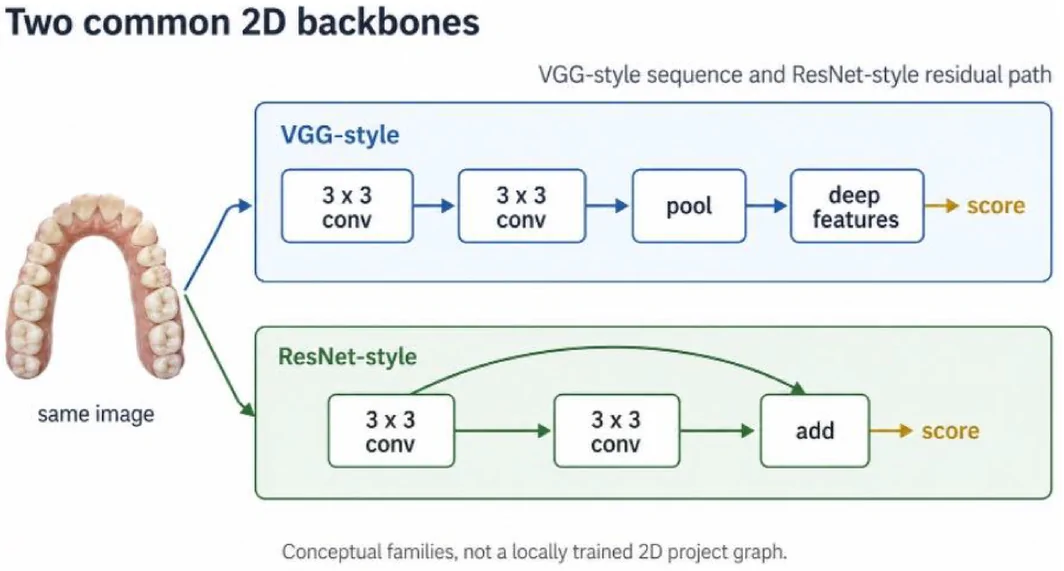
Two common two‐dimensional CNN backbones for crowding assessment. The same standardized occlusal image may enter a sequential VGG‐style stack or a residual ResNet‐style pathway. Both produce a learned representation that a task head can map to a crowding score. The schematic compares architecture families rather than one locally trained model [40, 42, 43].

#### 3.2.2 From Scan Segmentation to Point‐Cloud Prediction

A raw intraoral scan is typically represented as a three‐dimensional surface mesh that can be converted into a point‐cloud representation for analysis. In the present pipeline, a separately trained tooth‐segmentation model is first applied to the scan; background (gingival tissues) is removed, the segmented dental surface is standardized, and 1500 mesh‐cell centers are sampled, with their three‐dimensional coordinates and surface‐normal features forming the point‐cloud input. PointNet or PointNet++ then analyzes this three‐dimensional geometry to generate an arch‐level estimate of crowding. The complete workflow is therefore scanning acquisition → tooth segmentation → point‐cloud construction → crowding prediction [39, 44, 45, 46, 47, 48, 49].

Segmentation and prediction are separate tasks. The segmentation model identifies the relevant dental structures and removes irrelevant or non‐dental surfaces. The PointNet models receive the pre‐segmented dental geometry and estimate crowding. Accurate segmentation is therefore an upstream requirement for this pipeline.

Previous photograph‐based examples have linked landmark or boundary detection to a crowding calculation [40, 41], whereas the three‐dimensional intraoral‐scan studies cited here evaluate tooth segmentation and labeling as separate tasks [44, 45, 46, 47, 48, 49]. A jointly trained raw‐scan‐to‐crowding system was not evaluated in the present pipeline. The stages remain separate because tooth‐level segmentation and arch‐level crowding require different annotations, and the available cohort was not assembled for joint supervision. This modular design also provides a practical advantage: segmentation quality and prediction error can, in principle, be examined separately, although segmentation performance was not independently evaluated in the present cohort.

This modular workflow introduces a chain of potential errors. Scan acquisition can omit or distort narrow interproximal surfaces; segmentation can merge adjacent crowded teeth or misplace tooth boundaries; mesh cleanup can change triangles and surface normals; and sampling 1500 cell centers can discard small local structures. Coordinate normalization and neighborhood scale can further change which points the model treats as nearby [50]. Errors introduced at any of these stages can propagate to the final prediction. The authors' pilot investigations therefore evaluate crowding prediction conditional on the processed meshes, not the reliability of an entire raw‐scan‐to‐output system. Three‐dimensional input also does not resolve the target problem: the model still learns whichever clinician rating or software‐defined measurement is used as its label.

#### 3.2.3 PointNet and PointNet++

A point cloud is an unordered set of three‐dimensional samples, so the order in which the points are stored should not affect the model's prediction. PointNet applies the same feature network to every point and then uses a symmetric global max‐pooling operation to combine the most informative features into a single representation of the entire dental arch [51]. In clinical terms, the model evaluates each sampled point independently and then identifies the strongest geometric signals that may be relevant to the overall arch‐level pattern. This design is computationally efficient and inherently insensitive to point ordering, making it well suited to three‐dimensional dental data. However, PointNet does not explicitly examine how neighboring points relate to one another or how local geometric features are organized in space (Figure 5). This limitation is particularly relevant in orthodontic applications, where local relationships—such as the position and orientation of adjacent teeth—can contribute substantially to the overall assessment of crowding.

**FIGURE 5:**
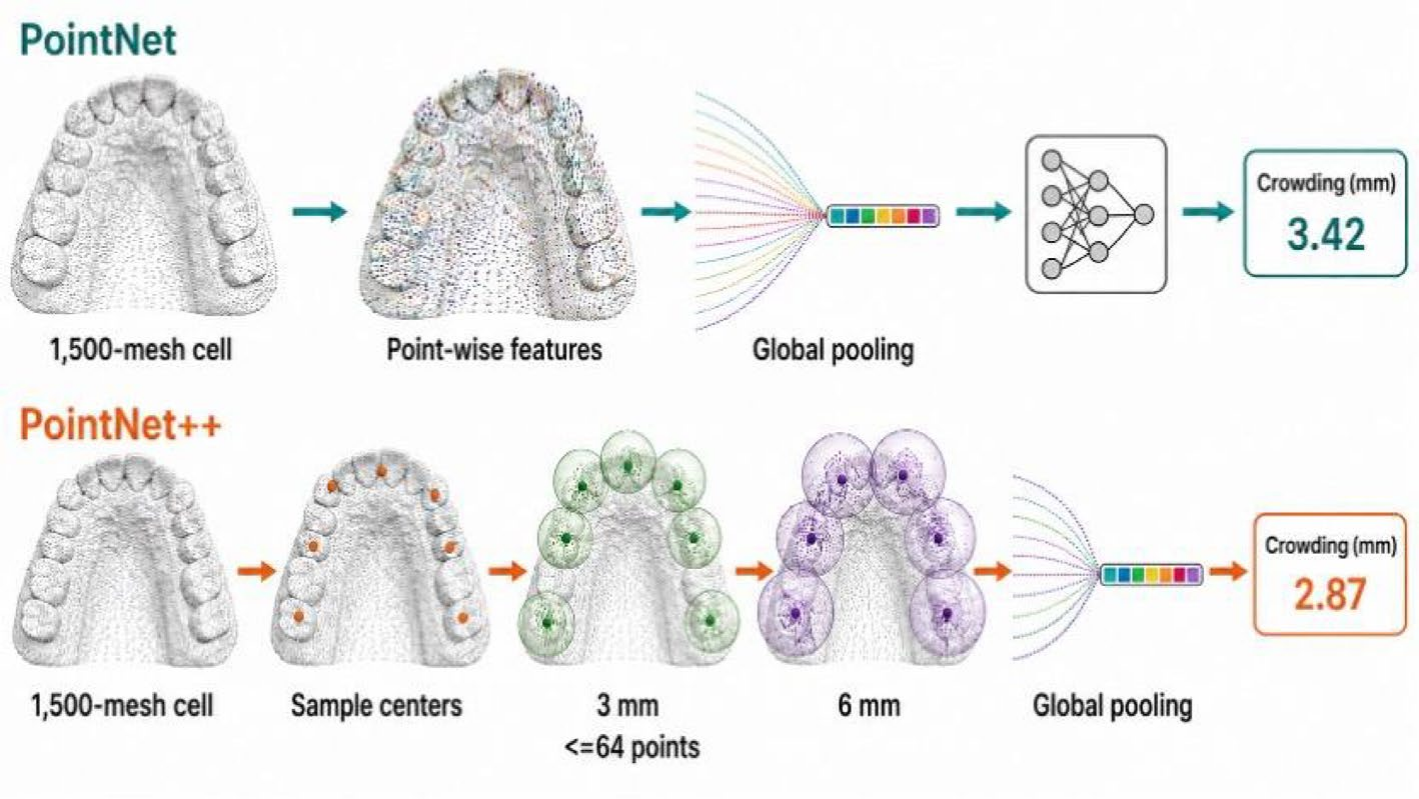
Conceptual comparison of PointNet and PointNet++ for arch‐level crowding regression. PointNet applies shared point‐wise features and global pooling to the full 1500‐point arch. PointNet++ samples representative centers and learns local features in 3 and 6‐mm three‐dimensional neighborhoods, retaining at most 64 points per neighborhood before global pooling. The numeric readouts are illustrative examples, not study results [50, 51].

PointNet++ (sometimes implemented or referred to as PointNet2) adds a hierarchical representation of local geometry [50]. It samples representative centers, groups nearby points around each center, learns a neighborhood feature, and repeats the process at a broader scale before global pooling. This structure is relevant to crowding because tooth rotation, overlap, interproximal relationships, and adjacent‐tooth positioning are local geometric patterns that can be captured through neighborhood‐based features (Figure 5).

In a companion study for dental crowding, the authors compared PointNet++ with PointNet using 495 arches from 284 patients and the unweighted mean of four expert ratings as the reference value. Both models generated a single arch‐level crowding estimate in millimeters. PointNet++ achieved an *R* ^2^ of 0.718 and an MAE of 1.54 mm, compared with an *R* ^2^ of 0.477 and an MAE of 2.16 mm for PointNet [39]. A separate patient‐disjoint analysis using a different evaluation protocol and repeated across five training seeds showed the same performance trend, with lower MAE and higher R^2^ for PointNet++ than for PointNet (Table 1).

**TABLE 1:** Patient‐disjoint PointNet and PointNet++ results for 495 arches from 284 patients using centered millimeter coordinates.

Split	Model	Radius	MAE (mm)	*R* ^2^	Grade accuracy
Patient‐disjoint 3‐fold; 5 seeds	PointNet	N/A	1.860 ± 0.052	0.619 ± 0.024	57.41% ± 2.01
Patient‐disjoint 3‐fold; 5 seeds	PointNet++	3/6 mm	1.530 ± 0.014	0.733 ± 0.004	65.13% ± 0.79

*Note:* MAE is the mean absolute error in millimeters (lower is better); *R* ^2^ is the proportion of outcome variation explained by the model (higher is better, but it is not percentage accuracy). Grade accuracy is the percentage of arches for which the predicted and reference values fall within the same severity interval among six predefined intervals, with cut points at −8, −4, 0, 4, and 8 mm. Values are mean ± standard deviation across five training seeds.

### 3.3 Clinician‐Aware Agentic AI

A three‐dimensional model represents measurable dental geometry. However, prediction errors may be greater in clinically complex cases involving substantial tooth rotation or inclination, blocked‐out or missing teeth, asymmetry, a pronounced curve of Spee, or severe crowding. These error sources are related but not identical. A clinician‐aware system should therefore distinguish geometric model outputs from contextual clinical observations and preserve the provenance of each source [52, 53].

#### 3.3.1 Why an Agent Can Add Value

An agent can provide a common interface for structured measurements, free‐text clinical observations, and model outputs, while recording where each item came from [53, 54]. It can compare sources, identify disagreement, and decide whether the available evidence is sufficient for a bounded answer. ReAct and Toolformer are general‐purpose examples in which a language model alternates between reasoning and calls to external tools such as search systems or calculators [55, 56].

A future AI system could receive a three‐dimensional scan, image, clinician note, measurement report, or record‐derived finding as separate evidence. Uncertainty estimates could route poorly supported cases for expert review [57], and active learning could prioritize cases whose labels are expected to improve the model [58]. These capabilities are proposed research directions rather than evaluated components of the present review.

A simple question is raised: can an Agent use a short verbal description to improve a three‐dimensional estimate? To examine this question, the Agent combines a legacy PointNet++ prediction with the description. Based on the verbal information, Gemini selects one of five predefined numerical adjustments (−2, −1, 0, +1, or +2 mm), which is then applied to the PointNet++ estimate by deterministic code. The Agent can therefore interpret flexible clinical language, while its numerical action remains bounded and directly inspectable, and the downstream fusion step is deterministic (Figure 6).

**FIGURE 6:**
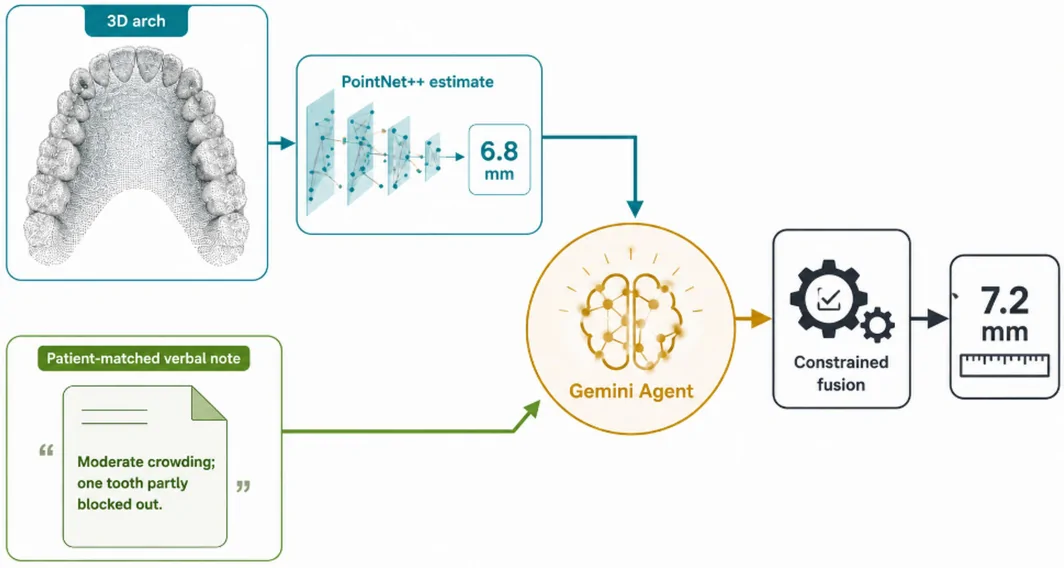
Agent‐plus‐verbal pathway used in the retrospective demonstration. A patient‐out‐of‐fold prediction from the legacy PointNet++ model and one matched, record‐derived verbal description enter Gemini 2.5 Flash‐Lite. Gemini selects a bounded adjustment and deterministic constrained fusion returns the final crowding estimate. The displayed 6.8‐ and 7.2‐mm values illustrate the calculation path rather than aggregate study results.

#### 3.3.2 Minimal Agent‐Plus‐Verbal Demonstration

The retrospective demonstration included 495 arches from 284 patients in three patient‐disjoint folds. The legacy PointNet++ model provided the three‐dimensional estimate, and Gemini 2.5 Flash‐Lite processed one matched, record‐derived description per arch. For example, one arch input stated, “Maxillary right canine is described as rotated. Blocked‐out and missing teeth were noted.” The descriptive wording was template‐generated from stored clinical annotation fields. Results are given in Table 2.

**TABLE 2:** Minimal agent‐plus‐verbal demonstration.

Method	Verbal input	MAE	Grade accuracy
Legacy 3D PointNet2 (0.2/0.4 mm; uncentered) only	None	1.951 mm	61.21%
+Gemini Agent + verbal	Matched patient	1.437 mm	65.86%
+Gemini Agent +verbal	Wrong patient	3.067 mm	47.07%

*Note:* All rows use the same 495‐arch, patient‐disjoint protocol and the legacy PointNet2 model with 0.2/0.4‐mm radii on uncentered coordinates. The wrong‐patient row tests whether matching the description to the correct patient matters. This is a separate experiment from the centered five‐seed comparison in Table 1.

The reference outcome was the unweighted mean of the four manual expert ratings. MAE is the average absolute distance between a prediction and that reference in millimeters; lower is better. Grade accuracy uses the same six intervals defined in Section 3.2.3; higher is better.

Matched verbal input reduced MAE from 1.951 to 1.437 mm and increased grade accuracy from 61.21% to 65.86%. When descriptions were assigned to the wrong patients, MAE worsened to 3.067 mm and grade accuracy fell to 47.07%. In this retrospective demonstration, matched record‐derived descriptions were associated with improved prediction, whereas mismatched information degraded performance.

#### 3.3.3 Interpretation Boundary

This result illustrates the retrospective feasibility of using a bounded Agent to combine a 3D predictive model with a matched verbal description and provide a traceable final calculation.

The descriptions were generated retrospectively from existing record‐derived annotations, including a historical severity field; they were not prospectively collected chairside clinician comments. Future prospective studies should collect standardized clinician observations independently, before the reference crowding score is established, to reduce the risk of information leakage between the verbal input and the evaluation target.

## 4 Limitations and Research Priorities

The quantitative demonstrations presented in this review are retrospective and derived from a single cohort. Two‐dimensional models remain sensitive to projection and image quality, while three‐dimensional models depend on scan quality, segmentation, and point‐cloud processing. External validation is needed before either approach can be generalized [59, 60].

The choice of reference standard is also important. The expert‐consensus ratings provide a practical reference, but crowding can also be defined by space discrepancy, alignment irregularity, three‐dimensional displacement, or clinical severity. Future studies should report the label definition and rater agreement together with model performance.

Future three‐dimensional studies should test tooth‐aware or contact‐aware neighborhoods and intermediate geometric targets such as tooth width, personalized arch perimeter, or local displacement. These components may improve both prediction and interpretation.

Future Agent studies should prospectively collect standardized clinician observations independently of the reference assessment and compare 3D‐only prediction with matched, missing, and mismatched verbal input. They should also test whether uncertainty‐based review and active sample selection reduce large errors or labeling effort.

Together, these directions extend the Agent from a fixed two‐input demonstration to a system that can determine when the 3D model provides sufficient evidence, when to use human information, and when to request review.

## 5 Summary

Dental crowding is not a single, uniform clinical concept. It can be described using several related but nonidentical measures, including tooth‐size–arch‐length discrepancy, alignment irregularity, three‐dimensional tooth displacement, and clinician‐assigned severity. Digital models provide more consistent access to three‐dimensional dental anatomy and facilitate repeatable measurements, while two‐dimensional CNNs learn from visual features available in clinical photographs. PointNet‐based models, in contrast, learn directly from three‐dimensional dental surface representations, although their performance remains dependent on the quality of segmentation and the clinical or software‐defined labels used for training.

The main three‐dimensional finding is that PointNet++ outperformed PointNet for crowding prediction, supporting the value of explicitly modeling local neighborhood geometry. In a separate Agent demonstration using legacy PointNet++ predictions under a different analysis setting, adding a matched record‐derived description reduced MAE from 1.951 to 1.437 mm, whereas using information from the wrong patient increased MAE to 3.067 mm. These findings should be interpreted as preliminary and complementary rather than as evidence from a single unified model or validation cohort. Together, the new approach suggests a potential future direction: combining physically grounded three‐dimensional geometric modeling with flexible and traceable patient‐specific clinical information, followed by prospective validation of the complete workflow in real clinical settings.

## Conflicts of Interest

All authors declare no conflicts of interest.

## Data Availability

Research data are not shared.
